# Disease‐induced and treatment‐induced alterations in body composition in locally advanced head and neck squamous cell carcinoma

**DOI:** 10.1002/jcsm.12487

**Published:** 2019-09-19

**Authors:** Anna C.H. Willemsen, Ann Hoeben, Roy I. Lalisang, Ardy Van Helvoort, Frederik W.R. Wesseling, Frank Hoebers, Laura W.J. Baijens, Annemie M.W.J. Schols

**Affiliations:** ^1^ Division of Medical Oncology, Department of Internal Medicine Maastricht University Medical Center+ Maastricht The Netherlands; ^2^ GROW‐School of Oncology and Developmental Biology Maastricht University Medical Center+ Maastricht The Netherlands; ^3^ Department of Respiratory Medicine, NUTRIM School of Nutrition and Translational Research in Metabolism Maastricht University Medical Center+ Maastricht The Netherlands; ^4^ Danone Nutricia Research Nutricia Advanced Medical Nutrition Utrecht The Netherlands; ^5^ Department of Radiation Oncology MAASTRO Clinic Maastricht The Netherlands; ^6^ Department of Otorhinolaryngology, Head & Neck Surgery Maastricht University Medical Center+ Maastricht The Netherlands

**Keywords:** Muscle wasting, Cancer cachexia, Head and neck, Chemoradiation, Bioradiation, Tube feeding

## Abstract

**Background:**

Chemoradiation or bioradiation treatment (CRT/BRT) of locally advanced head and neck squamous cell carcinoma (LAHNSCC) comes with high toxicity rates, often leading to temporary tube feeding (TF) dependency. Cachexia is a common problem in LAHNSCC. Yet changes in body composition and muscle weakness during CRT/BRT are underexplored. Strong evidence on the effect of TF on body composition during treatment is lacking. The aim of this cohort study was to assess (i) the relationship of fat‐free mass index (FFMI) and handgrip strength (HGS) with CRT/BRT toxicity and outcome, (ii) body composition in patients treated with chemoradiation (cisplatin) vs. bioradiation (cetuximab), and (iii) the effect of the current TF regime on body composition and muscle strength.

**Methods:**

Locally advanced head and neck squamous cell carcinoma patients treated with CRT/BRT between January 2013 and December 2016 were included (*n =* 137). Baseline measurements of body composition (bioelectrical impedance analysis) and HGS were performed. Toxicity grades (Common Terminology Criteria for Adverse Events) were scored. In a subset of 69 patients, weight loss, body composition, and HGS were additionally assessed during and after CRT/BRT. TF was initiated according to the Dutch guidelines for malnutrition.

**Results:**

In this cohort (68% male, mean age 59 ± 8 years), the incidence of baseline muscle wasting, defined as FFMI < P_10_, was 29%. Muscle wasting was present in 23 of 100 (23%) chemoradiation patients and 17 of 37 (46%) bioradiation patients (*P* = 0.009). Muscle‐wasted patients required more unplanned hospitalizations during CRT (*P* = 0.035). In the chemoradiation subset, dose‐limiting toxicity was significantly higher in wasted vs. non‐wasted patients (57% vs. 25%, *P* = 0.004). Median follow‐up was 32 months. Multivariate Cox regression analysis identified muscle wasting as independent unfavourable prognostic factor for overall survival [hazard ratio 2.1 (95% CI 1.1–4.1), *P* = 0.022] and cisplatin as favourable prognostic factor [hazard ratio 0.3 (95% CI 0.2–0.6), *P* = 0.001]. Weight and HGS significantly decreased during CRT/BRT, −3.7 ± 3.5 kg (*P* < 0.001) and −3.1 ± 6.0 kg (*P* < 0.001), respectively. Sixty‐four per cent of the patients required TF 21 days (range 0–59) after CRT/BRT initiation. Total weight loss during CRT/BRT was significantly (*P* = 0.007) higher in the total oral diet group (5.5 ± 3.7 kg) compared with the TF group (3.0 ± 3.2 kg). Loss of FFM and HGS was similar in both groups.

**Conclusions:**

In LAHNSCC patients undergoing CRT/BRT, FFMI < P_10_ is an unfavourable prognostic factor for overall survival, treatment toxicity, and tolerance. Patients experience significant weight and FFM loss during treatment. Current TF regime attenuates weight loss but does not overcome loss of muscle mass and function during therapy. Future interventions should consider nutritional intake and additional strategies specifically targeting metabolism, loss of muscle mass, and function.

## Introduction

Patients suffering from advanced cancer often develop cachexia, a multifactorial syndrome with unintended loss of skeletal muscle mass, caused by a variable combination of reduced food intake and changes in metabolic processes.^1^ In head and neck cancer (HNC) patients, the prevalence of cachexia is 3–52% at diagnosis, depending on tumour location and stage.^2^, ^3^ Resection of tumours in the head and neck region can be truly mutilating, preventing sufficient oral intake, which can lead to increased weight loss. Preparatory procedures for radiotherapy (RT), such as tooth extractions,^4^ also contribute to a more difficult oral intake. During post‐operative chemoradiation (CRT) or primary CRT or bioradiation treatment (BRT) of locally advanced head and neck squamous cell carcinoma (LAHNSCC), weight loss, in terms of reduction in fat mass (FM), fat‐free mass (FFM), or a combination of both, is induced even further owing to therapy‐related toxicity, also interfering with oral intake^5^ [mucositis, taste loss, oropharyngeal dysphagia (OD)] or putative catabolic effects on skeletal muscle mass.^6^, ^7^, ^8^

Low skeletal muscle mass in HNC patients is associated with increased (chemo)radiotherapy‐induced toxicity (e.g. mucositis, radiation dermatitis, neutropaenia, and nephrotoxicity); this leads to treatment interruptions causing decreased treatment efficacy and cure rates.^3^, ^9^ Furthermore, skeletal muscle mass loss during the course of RT has been associated with higher mortality rates.^10^

Therefore, assessment of body composition prior to and during treatment is of interest in LAHNSCC patients undergoing surgery and/or CRT/BRT to individually tailor interventions that optimize weight in general and muscle mass in particular. A body mass index (BMI) measurement alone cannot reveal a low muscle mass. Ideally, a rapid screening method for muscle mass such as bioelectrical impedance instead of more advanced imaging methods would be suitable for this purpose. Nowadays standard nutritional intervention includes the administration of tube feeding (TF) to stabilize weight loss when oral intake is impaired throughout the total course of HNC therapy.^11^ It is expected that TF partially limits loss in FM. However, optimizing and maintaining muscle mass might require additional anabolic and/or anti‐catabolic ingredients and/or interventions besides TF.

Yet strong evidence of the effect of TF on the exact course and composition of weight loss during therapy is lacking, limiting insight on recovery or cachexia prevention. Previous work has focused on long‐term weight loss (minimum 2–3 months after CRT/BRT completion).^12^ Short‐term changes in body composition, as well as differences in weight and muscle loss between patients receiving cetuximab vs. cisplatin as radiosensitizer during RT, have not yet been sufficiently studied.

The aim of this cohort study was to assess (i) the relationship of FFM index (FFMI) and handgrip strength (HGS) with CRT/BRT toxicity and outcome, (ii) changes in body composition in patients treated with chemoradiation (cisplatin) vs. bioradiation (cetuximab), and (iii) the effect of the current TF regime on body composition and muscle strength.

## Materials and Methods

### Study design and population

Patients with LAHNSCC, who were treated with CRT or BRT (as post‐operative or primary radiation treatment) in the Maastricht University Medical Center (MUMC+) and Maastro Clinic between January 2013 and December 2016, were included in this study. Patients were prospectively followed up as part of a larger prospective non‐interventional registration study for HNC patients treated with RT, CRT, or BRT, which was approved by the Institutional Review Board of Maastro Clinic (http://ClinicalTrials.gov Identifier: NCT01985984). Additional data were extracted from the medical patient files, with approval from the medical ethics committee of the MUMC+ according to the non‐WMO obligatory Medical Research Involving Human Subjects Act.^13^ All patients received primary chemoradiation or bioradiation (cisplatin or cetuximab, respectively) or adjuvant post‐operative chemoradiation (cisplatin) therapy with curative intent. Exclusion criteria were palliative treatment, oesophageal tumours, histology other than squamous cell carcinoma, no administration of systemic therapy, and age < 18 years.

### Oncological treatment

Cisplatin was administered intravenously on Days 1, 22, and 43, in doses of 100 mg/m^2^.^14^, ^15^ Cetuximab was indicated in patients not fit for cisplatin, for example, in case of prior cerebrovascular accidents, myocardial infarction, intermittent claudication, neuropathy, renal function loss, or pre‐existent severe hearing loss. A loading dose of 400 mg/m^2^ was administered intravenously 1 week before RT initiation, followed by 250 mg/m^2^ weekly during RT.^16^

For patients receiving definitive RT with concurrent cisplatin, intensity‐modulated RT was applied five times per week for 7 weeks in 35 daily fractions of 2 Gy to a total dose of 70 Gy in 47 days. Patients receiving cetuximab as part of definitive bioradiation received accelerated fractionated RT with twice‐daily fractions in the final week of RT to a total of 68 Gy in 34 fractions in 38 days. For patients undergoing adjuvant post‐operative chemoradiation, a total of 66 Gy in 35 fractions over 45 days was administered concurrently with cisplatin.

### Nutritional treatment

Tube feeding was started when patients met the criteria described in the Dutch guidelines for malnutrition.^17^ All patients were screened and counselled on a weekly basis by a dietician for nutritional status and requirements for their support plan, in brief: patients who reach 75–100% of their nutritional requirements received protein and energy‐enriched/fortified main meals and between meal snacks and if required oral nutritional supplements (ONS). The support plans were monitored and adjusted if required. Patients with intake between 50% and 75% of the calculated nutritional requirements were initially advised to use ONS or TF in addition to daily oral intake. When intake was <50% of the calculated nutritional need, full TF was indicated, supplemented with any possible safe oral intake. Patients were stimulated to practise swallowing in order to maintain oropharyngeal function.^18^ TF was administered through a nasogastric tube or gastrostomy, with the latter either as a percutaneous endoscopic gastrostomy or a percutaneous radiological gastrostomy.

### Measurements

Weight was measured weekly before and during treatment at the standard visits to the dietician, medical oncologist, and radiation oncologist. Height was measured only once at baseline. Body composition was determined by bioelectrical impedance analysis (BIA) using an Omron device, model BF306 (OMRON Healthcare Group, Hoofddorp, The Netherlands). A Jamar hydraulic hand dynamometer was used to measure grip strength (JA Preston Corporation, Jackson, MI, USA). The highest value of three measurements on both hands was noted. HGS values of the dominant hand were then binary divided in normal and low grip strength with a cut‐off value based on the 10th percentile reference values described by Spruit et al.^19^ Pre‐RT weight loss was patient reported by asking whether and how much weight was unintentionally lost during the previous months. As pretreatment weight loss was only patient reported, it was decided to define muscle wasting on the basis of an FFMI < 17 (for men) or <15 kg/m^2^ (for women), based on reference values of the 10th percentile in Caucasians.^20^

### Statistical analyses

All statistical analyses were performed using IBM SPSS Statistics for Windows, Version 25 (IBM Corp., Armonk, New York, USA). Descriptive statistics were reported in frequency distributions and absolute numbers by using independent samples *t*‐test and *χ* ^2^ test. Paired samples *t*‐test was used for the determination of mass loss. Kaplan–Meier was performed with log rank (Mantel–Cox). Univariate Cox regression was performed, and subsequently, multivariate Cox regression was carried out by means of backward log rank to plot overall survival (OS). Significance was assumed in case *P* < 0.05. In multivariate Cox regression, probability for stepwise removal was set at 0.10.

## Results

### Disease‐induced muscle wasting

Between 2013 and 2016, 192 patients with LAHNSCC were treated with CRT/BRT. In 137 cases, body composition measurements (BIA) at baseline (pre‐RT) were collected, and this cohort represents the population of the current analysis. In 69 of these patients, additional measurements were collected in weeks 3 and 4 of treatment and 1–2 weeks after CRT/BRT completion.

At the start of CRT/BRT, 40 out of 137 patients (29%) met the criteria for muscle wasting on the basis of an FFMI < P10.^21^ These patients were also characterized by a lower World Health Organization performance status, lower HGS, and a higher incidence of OD ≥ Grade 2 according to the Common Terminology Criteria for Adverse Events Version 4.0 (CTCAE) (*Table* 1). Muscle wasting was not prevalent in patients receiving adjuvant CRT than in patients receiving primary CRT/BRT (19% vs. 32%, respectively, *P* = 0.138). When evaluating the cisplatin subgroup only, the incidence of muscle wasting did not significantly differ between patients starting primary and post‐operative CRT (26% vs. 15% respectively, *P* = 0.283). The presence of OD was significantly higher in patients with oropharyngeal or oral cavity tumours compared with other tumour sites (36% vs. 16% respectively, *P* = 0.012) and was significantly higher in patients who underwent surgery and post‐operative CRT compared with primary CRT/BRT (41% vs. 23% respectively, *P* = 0.047). T‐stage did not significantly differ between patients with and without OD, nor between the wasted and non‐wasted patients.

**Table 1:** Baseline characteristics—normal fat‐free mass index vs. fat‐free mass index < 10th percentile (P_10_)

Variables	Baseline group (*n =* 137)		
	Normal FFMI, *n =* 97 (71%)	FFMI < P_10_, *n =* 40 (29%)	*P*‐value
Age (years)	59.2 ± 7.3	59.6 ± 8.2	0.769^a^
Sex			
Male	71 (73%)	22 (55%)	
Female	26 (27%)	18 (45%)	**0.038** b
BMI (kg/m^2^)	27.0 ± 3.9	19.6 ± 2.0	**<0.001** a
Mean pretreatment weight loss (%)	2.6 ± 4.3	3.8 ± 5.1	0.158^a^
CTCAE OD ≥ grade 2 at start RT			
Yes	18 (19%)	19 (48%)	
No	79 (81%)	21 (52%)	**0.001** b
Tobacco use			
Yes	87 (90%)	38 (95%)	
No	10 (10%)	2 (5%)	0.318^b^
Alcohol consumption of at least 1 per day			
Yes	55 (57%)	26 (65%)	
No	42 (43%)	14 (35%)	0.369^b^
WHO performance status			
0	19 (20%)	1 (3%)	
1	75 (77%)	37 (93%)	
2	3 (3%)	2 (5%)	**0.034** b
Handgrip strength ( kg)			
Male	47 ± 11	38 ± 8	**<0.001** a
Female	29 ± 5	24 ± 5	**0.043** a
Primary tumour site			
Nasopharynx	5 (5%)	2 (5%)	
Oropharynx	39 (41%)	14 (30%)	
Hypopharynx	12 (13%)	7 (19%)	
Oral cavity	17 (16%)	5 (14%)	
Larynx	20 (21%)	11 (30%)	
Unknown primary	2 (2%)	1 (3%)	
Other	2 (2%)	0 (0%)	0.854^b^
T classification			
Tx	3 (3%)	1 (3%)	
T0	5 (5%)	0 (0%)	
T1	13 (14%)	2 (5%)	
T2	19 (20%)	7 (16%)	
T3	23 (24%)	14 (35%)	
T4	34 (34%)	16 (41%)	0.356^b^
N classification			
N0	15 (15%)	11 (30%)	
N1	14 (14%)	2 (5%)	
N2	66 (69%)	25 (60%)	
N3	2 (2%)	2 (5%)	0.152^b^
Tumour stage			
Stage II–III	17 (17%)	7 (19%)	
Stage IV	80 (83%)	32 (81%)	0.997^b^
P16			
P16+ oropharynx	24 (25%)	3 (8%)	
Others	73 (75%)	36^c^ (92%)	**0.024** b
CRT timing			
Primary	71 (75%)	34 (86 %)	
Adjuvant	26 (25%)	6 (14%)	0.138^b^
Systemic therapy			
Cisplatin	77 (80%)	23 (57%)	
Cetuximab	20 (20%)	17 (43%)	**0.009** b
Radiotherapy on neck			
Unilateral	7 (7%)	1 (3%)	
Bilateral	89 (92%)	39 (97%)	
No neck RT	1 (1%)	0 (0%)	0.451^b^
Tube feeding administration			
Yes	60 (62%)	28 (70%)	
No	37 (38%)	12 (30%)	0.366^b^
Type of feeding tube			
No feeding tube	26 (27%)	6 (15%)	
NGT only	7 (7%)	1 (3%)	
PEG	8^d^ (8%)	3 (8%)	
PRG	56^e^ (58%)	30^f^ (75%)	0.247^b^

Bold values denote statistical significance at the *P* < 0.050 level.

BMI, body mass index; CRT, chemoradiation; NGT, nasogastric tube; OD, oropharyngeal dysphagia (Common Terminology Criteria for Adverse Events grade 2 OD or higher [CTCAE]); PEG, percutaneous endoscopic gastrostomy; PRG, percutaneous radiologic gastrostomy; RT, radiotherapy; Tumour, nodes, and metastasis (TNM) classification 7th edition.^78^ Owing to rounding off, percentages may not count up to exactly 100; WHO, World Health Organisation.

a Independent samples *t*‐test.

b *χ* ^2^.

c One missing.

d One patient did not use feeding tube.

e Ten patients did not use feeding tube.

f Six patients did not use feeding tube.

In patients receiving cetuximab, a significantly larger proportion of patients had an FFMI < P_10_ than had patients receiving cisplatin (46% vs. 23%, *P* = 0.009), but this was not reflected in significant differences in BMI (24.9 ± 4.4 vs. 24.5 ± 5.9 kg/m^2^, *P* = 0.629). Cetuximab patients more often showed CTCAE OD ≥ Grade 2 at the start of CRT/BRT and had significantly higher levels of tobacco and alcohol use (*Table* A1). No significant difference was found in World Health Organization performance status between cisplatin receivers and cetuximab receivers (*P* = 0.119).

### Treatment‐induced changes in body composition

Information on body composition and grip strength throughout the course of CRT/BRT was available in 69 patients. Baseline characteristics in this subset were comparable with those of the total cohort of 137 patients shown in *Table* 1.

The incidence of muscle wasting at baseline in the subgroup of 69 patients was 20 of 69 (29%), comparable with the incidence rate of the total group. The incidence of muscle wasting increased to 25 of 69 (36%) at the end of CRT/BRT. Seven patients with a normal FFMI (14%) reached FFMI < P_10_ during or at the end of CRT/BRT (four received TF), and two muscle‐wasted patients (10%) had a normal FFMI after CRT/BRT completion. Both of them used additional TF.

The mean weight loss over the course of CRT/BRT was 3.7 ± 3.5 kg (*P* < 0.001) in which FFM covered 1.8 ± 3.7 kg and FM 1.9 ± 3.1 kg. Also, HGS significantly decreased during treatment by 3.1 ± 6.0 kg (*P* < 0.001). Dividing the population in a TF (*n* = 48) and total oral diet (TOD) (*n =* 21) group, the total weight loss throughout CRT/BRT was significantly higher in the TOD group when compared with the TF group: 5.5 ± 3.7 and 3.0 ± 3.2 kg, respectively (*P* = 0.007). FM and FFM decreased significantly in both subgroups. In addition, HGS decreased by 3.1 ± 5.4 kg (*P* < 0.001) in the TF subgroup and by 3.0 ± 7.2 kg (*P* = 0.067) in the TOD subgroup. Specification of weight loss and HGS is shown in *Table* A2.

Tube feeding was initiated at a median of 21 days (range 0–59) after the first RT fraction. Despite this nutritional support, patients receiving TF continued to lose weight (1.7 ± 2.8 kg, *P* < 0.001) in both FM (0.8 ± 3.5 kg, *P* = 0.112) and FFM (0.9 ± 3.2 kg, *P* = 0.054) and lost HGS significantly during the course of treatment (3.1 ± 5.4 kg, *P* < 0.001). Full details of mass and function loss are available in the Appendix. When investigating cisplatin and cetuximab receivers separately, the mean weight loss throughout the course of CRT/BRT (from RT start up to 2 weeks after CRT/BRT completion) was 4.1 ± 3.7 (*P* < 0.001) and 2.7 ± 3.0 kg (*P* < 0.002), respectively. The in‐between group difference was not statistically significant (*P* = 0.184).

When comparing changes in body composition between patients with a prophylactically inserted feeding tube (*n =* 41, inserted before start of first RT or within 7 days after RT initiation) with patients with reactively inserted feeding tubes (*n =* 7), no statistically significant differences could be shown. However, total weight loss throughout the course of therapy tends to be higher in the subgroup with reactively inserted gastrostomies when compared with prophylactic gastrostomies: 4.8 ± 2.6 vs. 2.7 ± 3.3 kg, respectively (*P* = 0.118).

### Muscle wasting and side effects of chemoradiation or bioradiation treatment

Eighteen out of 40 muscle‐wasted patients (45%) at the start of treatment did not complete CRT/BRT as planned, owing to scheme changes such as treatment interruptions, dose reductions, and postponement or adjustment of RT or chemotherapy administration. These treatment changes were significantly (*P* = 0.019) more frequent than in the non‐wasted patients (25%).

Hematologic toxicity, ototoxicity, and renal failure were only determined in the population who received cisplatin as radiosensitizer, because cetuximab is not myelosuppressive and less nephrotoxic and neurotoxic than cisplatin.^22^ Overall dose‐limiting toxicity (including neutropaenia, renal failure, ototoxicity, etc.) was significantly higher in muscle‐wasted (57%) compared with non‐wasted (25%) patients (*P* = 0.004). (Specification in *Table* A3.)

Mean cumulative doses of administrated cisplatin significantly differed between the muscle‐wasted and non‐wasted population, namely, 230 vs. 268 mg/m^2^, respectively (*P* = 0.011). However, only three patients received <200 mg/m^2^, considering the effective cumulative dose (two non‐wasted and one wasted).^23^

Furthermore, from the 137 patients, 53 were additionally admitted to the hospital for reasons other than the planned admissions during CRT/BRT. Patients receiving cisplatin required significantly more additional hospital admissions than did patients receiving cetuximab: 48% vs. 14%, respectively (*P* < 0.001).

The incidence of unplanned hospitalizations tends to be higher in non‐wasted patients who received cisplatin; 40 out of 70 (52%) non‐wasted and 8 out of 23 (35%) wasted patients had unplanned admissions (*P* = 0.148). The mean additional days of hospital admissions for any reason in the total cohort of non‐wasted and wasted patients were 4.3 ± 6.8 and 2.3 ± 6.3, respectively (*P* = 0.112). Indications for hospitalization varied and included renal failure, dehydration, fever, obstipation, gastrostomy complications, nausea, and electrolyte imbalances. Reasons for hospitalization did not significantly differ between baseline muscle‐wasted and non‐wasted patients.

### Tube feeding

Eighty‐eight out of 137 (64%) patients were administered with TF during the course of CRT/BRT or within 30 days after the final fraction of RT. Sixty‐nine out of 100 (69%) cisplatin receivers became (temporarily) TF dependent, and 19 out of 37 (51%) of the cetuximab receivers required TF (*P* = 0.056). At 6 months after CRT/BRT completion, 15 out of 85 TF users (18%) were still TF dependent (one lost to follow‐up, one not reported, and one deceased). From these 15 subjects, four had post‐operative CRT, seven had muscle wasting at the start of CRT/BRT, and nine had CTCAE OD ≥ Grade 2 at the start of CRT/BRT.

### Muscle wasting as a predictor of overall survival

With the use of univariate Cox regression analysis, a negative prognostic value for OS was found for patients with baseline FFMI < P_10_, for patients with baseline BMI < 21 kg/m^2^, for patients with CTCAE OD ≥ Grade 2, and for patients receiving cetuximab as radiosensitizer vs. cisplatin. However, P16+ oropharyngeal tumours showed a positive prognostic value for OS. Multivariate Cox regression analysis showed an independent prognostic value for the variable FFMI < P_10_ and for type of systemic agent (*Table* 2).

**Table 2:** Univariate and multivariate Cox regression analyses of prognostic factors for overall survival in 137 locally advanced head and neck squamous cell carcinoma patients

Variable	Univariate analysis			Multivariate analysis^a^		
	HR	95% CI	*P*‐value	HR	95% CI	*P*‐value
Gender Male vs. female	0.941	0.487–1.818	0.858			
Age ≥60 vs. <60	0.706	0.381–1.306	0.267	0.543	0.285–1.035	0.064
WHO PS 1–2 vs. 0	3.941	0.950–16.359	0.059			
Baseline BMI <21 kg/m^2^ vs. higher	2.363	1.269–4.401	**0.007**			
Baseline FFMI <P_10_ vs. normal	2.907	1.574–5.368	**0.001**	2.090	1.083–4.035	**0.028**
Baseline OD CTCAE ≥ 2 vs. <2	3.177	1.717–5.880	**<0.001**	1.876	0.951–3.701	0.069
Tumour stage ≥Stage IV vs. <Stage IV	1.614	0.633–4.116	0.316			
P16+ oropharynx P16+ oropharynx vs. others	0.308	0.095–0.998	**0.0497**			
Indication for type of systemic agent Cetuximab vs. cisplatin	3.608	1.942–6.706	**<0.001**	3.322	1.682–6.560	**0.001**

Bold values denote statistical significance at the *P* < 0.050 level.

CI, confidence interval; CTCAE, Common Terminology Criteria for Adverse Events Version 4.0; FFMI, fat‐free mass index; HR, hazard ratio; OD, oropharyngeal dysphagia; WHO PS, World Health Organisation performance status.

a Backward log rank analysis.

**Figure 1:**
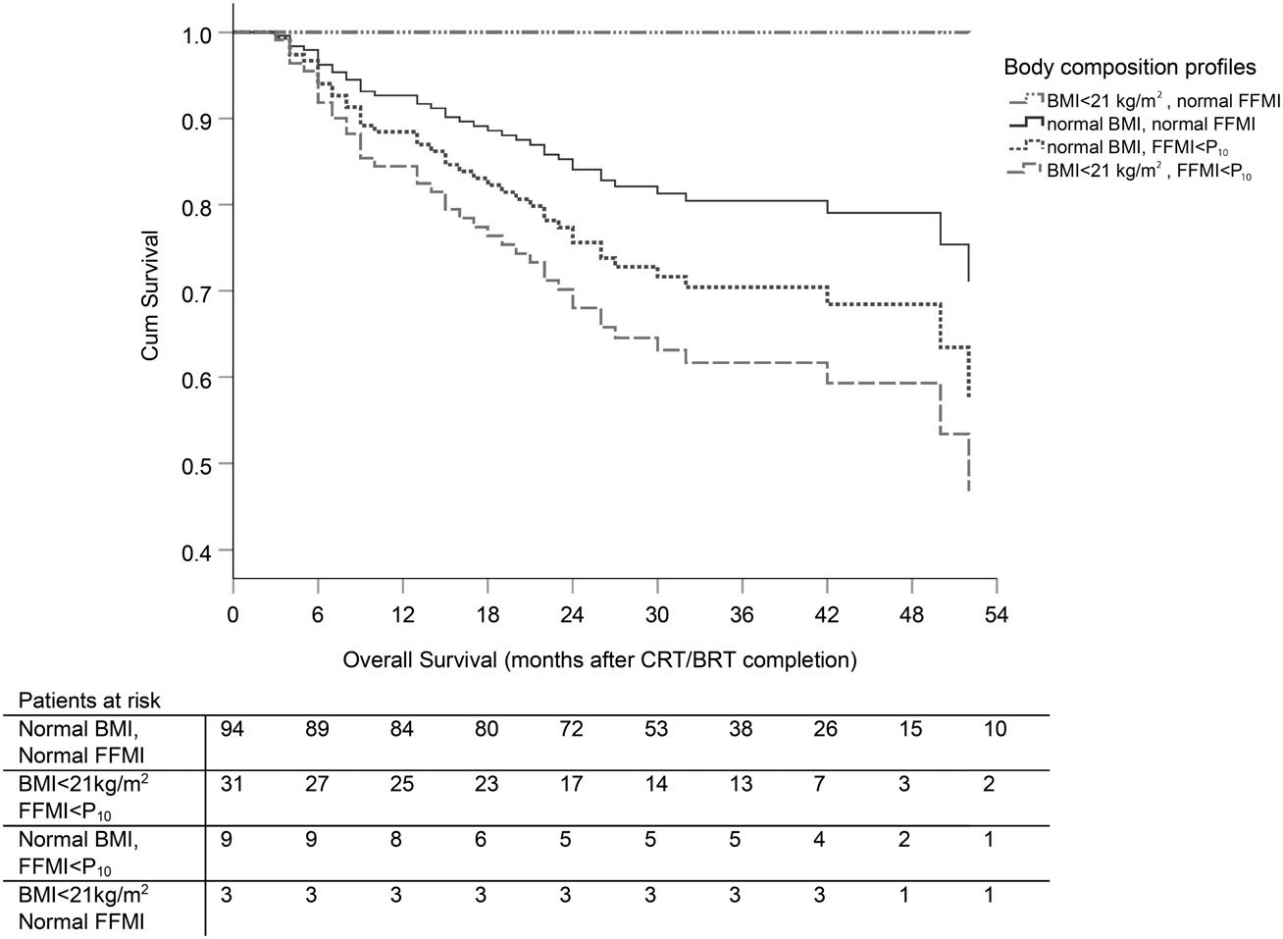
Multivariate Cox regression analyses for different body composition profiles, in which the following factors were taken into account: age < 60 (*P* = 0.078), CTCAE grade 2 OD at the start of CRT (*P* = 0.065), systemic therapy (cisplatin, cetuximab) (*P* = 0.001). BMI, body mass index < 21 kg/m^2^ 79; BRT, bioradiation treatment; CRT, chemoradiation; CTCAE, CTCAE, Common Terminology Criteria for Adverse Events Version 4.0; FFMI < P_10_, fat‐free mass index below 10th percentile; OD, oropharyngeal dysphagia.

*Figure* *1* shows the Cox regression survival plot of different body composition profiles. Patients with a low BMI and normal FFMI showed the best OS, and patients with a low BMI and FFMI < P_10_ showed the worst OS.

**Figure A1:**
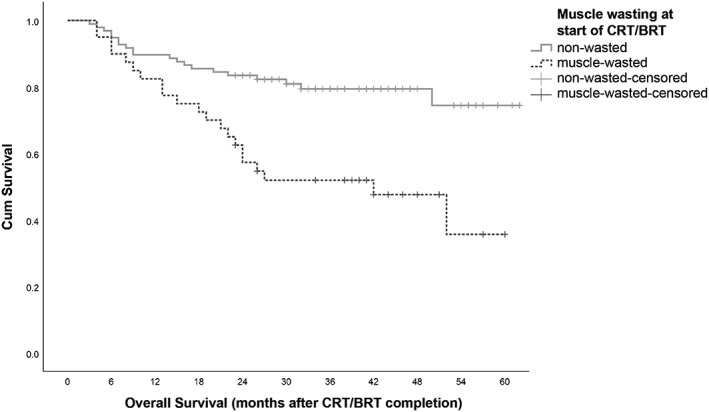
Kaplan–Meier survival plot—muscle‐wasted patients at the start of CRT/BRT vs. non‐wasted patients. *n* = 137, log rank (Mantel–Cox) significance *P* < 0.001. BRT, bioradiation treatment; CRT, chemoradiation.

At the time of censoring, 41 out of 137 patients (30%) were deceased. The median follow‐up was 32 months (range 3–62). OS for all patients was 75.9% at 2 years and 63.0% at 5 years. The OS rate at 2 and 5 years specified for muscle‐wasted patients was 57.3% and 35.7%, respectively. In non‐wasted patients, this OS rate was significantly higher, namely, 83.5% and 74.5% at 2 and 5 years, respectively [Kaplan–Meier, log rank (Mantel–Cox) significance *P* < 0.001, *Figure* *A1*].

In the 21 deceased muscle‐wasted patients, 12 had an LAHNSCC‐related death and five a non‐LAHNSCC‐related death, and in two patients, the cause of death was unknown. In the non‐wasted group, 19 patients died of disease progression, and in one case, the cause of death was unknown.

## Discussion

Owing to high rates of mucositis and OD limiting oral diet intake, weight loss during CRT/BRT in LAHNSCC patients seems almost inevitable despite current measures according to the Dutch guidelines for malnutrition. Furthermore, many patients already have a poor nutritional status at the start of treatment. Tumour‐induced OD and pre‐RT interventions such as tooth extractions and surgery may cause impaired oral intake in patients starting CRT/BRT.

In this Dutch patient cohort, an FFMI < P_10_ was found in 29% (*n =* 40/137) of the patients at the start of CRT, which is slightly higher than reported in present literature.^24^, ^25^ Kwon et al. reported a much lower pretreatment incidence of cachexia in Korean patients, namely, 6.1% (*n =* 22/361).^26^ These differences can probably partially be explained by the different diagnostic criteria that were used for muscle wasting and cachexia.^27^ In the present study, prediagnostic patient self‐report weight loss was considered insufficiently reliable to identify cachexia. Nevertheless, a cut‐off point of FFMI < P_10_ was considered appropriate to identify muscle wasting, as is recommended in the international guidelines.^20^, ^21^, ^28^ Especially in HNC, pretreatment muscle wasting is considered a multifactorial syndrome. Both tumour and patient characteristics may influence patient's oral intake and metabolism, leading to weight loss and muscle wasting.

One of the important factors influencing oral intake in LAHNSCC patients is the presence of OD. This is indeed reflected in the present and other study populations where cachectic patients have been shown to have OD significantly more often at diagnosis.^29^ OD increases the risk of malnutrition due to restrictive dietary adaptations made by the patient.^30^, ^31^ It has been suggested that OD is mainly caused by tumour invasion. However, T‐stage did not significantly differ between patients with and without OD. Nevertheless, primary tumour site was indeed significantly related to the presence of OD. The frequency of OD was significantly higher in patients with oropharyngeal or oral cavity tumours compared with other tumour sites (*P* = 0.012). In the HNC population, OD is usually caused by tumour‐related and treatment‐related anatomical and neurophysiological changes in the swallowing‐related structures (e.g. larynx, tongue, and pharynx) such as xerostomia, pharyngeal muscular fibrosis, decreased laryngeal sensation (RT‐induced and chemotherapy‐induced neuropathy), loss of laryngeal closure coordination, and trismus.^32^, ^33^, ^34^

Because cachexia is a muscle‐wasting syndrome, it would be likely that this muscle wasting also occurs in the swallowing muscles, thereby contributing to the development of OD. However, evidence supporting this theory is scarce.^35^

Another patient characteristic that significantly differed between wasted and non‐wasted subjects was gender. Despite different cut‐off points for male and female patients, a significantly higher proportion of muscle wasting was found in the female subjects. The present literature does not provide an explanation for this difference.^9^, ^10^, ^36^, ^37^ In the present cohort, a significant difference in the distribution of P16+ oropharyngeal tumours was observed between muscle‐wasted and non‐wasted patients. In the P16+ oropharyngeal tumour group, lower numbers of wasted patients were seen at the start of CRT/BRT. A plausible explanation might be that P16+ tumours are usually characterized by an advanced nodal stage and an early primary tumour stage.^38^, ^39^ A smaller primary tumour may cause less oral intake‐related problems than an advanced tumour stage does. In addition, patients with P16+ tumours are generally non‐smokers and non‐drinkers and presumably have a healthier lifestyle and less comorbidity compared to P16− patients.^40^

The present study also showed that pretreatment muscle wasting is an independent prognostic factor for OS in LAHNSCC patients. Kaplan–Meier and Cox regression analyses showed a significantly worse OS in patients with pretreatment muscle wasting, which is consistent with previous studies.^25^, ^26^, ^41^, ^42^, ^43^, ^44^, ^45^

This study is the first to evaluate the effect of different body ‘wasting' profiles in LAHNSCC on OS and the risk of misleading information when relying only on BMI. Although the sample size of the subgroups is small, *Figure* *1* suggests that a low BMI does not necessarily mean that patients are malnourished, nor that they are at risk for malnutrition‐related therapeutic consequences. The low BMI and normal FFMI subgroup (i.e. the lean ‘athletic' phenotype) even had the best OS outcome. More convincingly, there is a distinct difference in outcome between the two groups with normal BMI but FFMI < P_10_ vs. normal FFMI. These results highlight the importance of assessing body composition in the diagnostic trajectory and that even a simple tool such as BIA may provide clinically meaningful information.

Muscle wasting did influence the course of treatment, as a higher level of treatment toxicity was found in the muscle‐wasted group compared with the non‐wasted group. Muscle‐wasted patients receiving cisplatin had more often neutropaenia and renal failure interfering with HNC treatment. Higher numbers of early cessation of CRT in this group also reflect this. These higher frequencies of dose‐limiting toxicities are in line with previous results for LAHNSCC,^9^, ^44^, ^46^ and other cancer types.^47^, ^48^, ^49^ This finding demands for early identification of muscle wasting to allow personalized measures in order to obviate potential side effects of HNC treatment.

Unlike the side effects in the muscle‐wasted patient group, the need for additional hospital admissions tends to be higher in the non‐wasted group of cisplatin receivers. However, this remarkable finding was not statistically significant, and there is no clear explanation for this observation. Cisplatin is known for its high toxicity rates, especially nephrotoxicity requiring intravenous fluid administration to resolve,^22^ and this probably explains the difference in hospitalizations between cetuximab and cisplatin receivers. Besides muscle wasting, systemic therapy showed to be an independent predictor of OS, too, in favour of cisplatin. *Table* A1 on baseline characteristics shows a higher rate of FFMI < P_10_ in the cetuximab subgroup, suggesting a higher prevalence of comorbidity contraindicating cisplatin administration. Strikingly, this subgroup consisted mainly of patients with a normal BMI. In the Cox regression, patients receiving cetuximab showed significantly worse OS rates than did the cisplatin group.

Patients eligible for cisplatin appear to have better survival rates than patients requiring cetuximab due to contraindications for CRT.^50^, ^51^, ^52^, ^53^ In patients with comorbidity and muscle wasting, one can doubt the beneficial effect of bioradiation treatment relative to the high treatment burden. Based on the current results, conclusions cannot be drawn on whether or not muscle‐wasted patients with comorbidity can be treated with curative intent, but it does raise questions regarding current treatment protocols. Future studies should determine if the benefit of concurrent systemic therapy outweighs the increased toxicity in muscle‐wasted patients.

Despite the convincing impact of muscle wasting on OS, the current Dutch malnutrition guideline lacks standardized diagnostic and treatment strategies to tackle muscle wasting. Currently, clinicians try to influence overall weight loss by counselling and enriching the normal diet according to the Dutch guidelines, and in case of poor nutritional intake by administrating TF. This study shows that this strategy does not completely overcome the problem. The mean weight loss during the weeks of CRT was 3.7 ± 3.5 kg, of which FFM loss covered 47%. These findings differ slightly from two previous publications^54^, ^55^ in which weight loss during CRT, measured through dual‐energy X‐ray absorptiometry, was around 10 kg, of which 66–71% was lean body mass loss. However, the majority of the population in these studies were overweight or obese at the start of CRT, which has been shown to be linked to higher levels of weight loss during oncological treatment.^56^ The current results are, however, comparable with those of Atasoy et al.^57^ Despite TF administration, weight loss was still substantial and reached a mean of 3.0 ± 3.2 kg after CRT completion. However, this was significantly lower (*P* < 0.001) than the weight loss in the TOD group (5.5 ± 3.7 kg). Remarkably, the TOD group did not significantly lose weight during the first half of therapy but increasingly lost weight during the second half. A logical idea following these results would be to start TF prophylactically in the future. However, this strategy has been investigated by, among others, Brown et al.^58^ and did not show any beneficial effects on weight loss and health‐related quality of life.^59^ In spite of that, Brown showed that early TF can improve patient adherence to clinically indicated TF during treatment.^60^ Therefore, it would be a logical thought that prophylactic tube insertion at the beginning of CRT/BRT, before tube feeding is actually required, might lead to better patient adherence than in those receiving a feeding tube at the moment tube feeding is indicated (reactively). Our study was not designed to evaluate potential differences in prophylactic and reactive feeding tube insertion, but a trend could be objectified towards less weight loss in the prophylactic tube receivers when compared with the reactive group. On the basis of our study, we cannot comment on whether this difference in weight loss is due to a later TF initiation because of a wait‐and‐see attitude of the treating physician, owing to a poorer patient compliance or a combination of both.

On the other hand, the use of prophylactic feeding tubes has been argued because of potential harm to the long‐term swallowing function. Shune et al.^61^ hypothesized the ‘use it or lose it' principle: when the gastrostomy tube is used, oral intake is often reduced to a minimum, causing sensorimotor deprivation of the upper aerodigestive tract and pharyngeal constrictor muscle fibroses. This leads to deconditioning of the swallowing mechanism. However, the present literature is ambiguous on the relationship between prophylactic TF and long‐term OD.^18^, ^62^, ^63^, ^64^, ^65^, ^66^ Therefore, supplemental TF to maximize the chance to reach the nutritional target remains to be a regime that deserves validation.

Despite starting TF in the present population, patients still lost weight especially in the form of FFM, and TF did not prevent loss of function (HGS) either. This underlines the idea that muscle wasting in LAHNSCC is not a nutritional problem on its own but that it is accompanied by cancer‐related and therapy‐related metabolic and inflammatory processes that are involved in muscle wasting, energy metabolism, and weight loss, too. Consequently, it cannot be ruled out that the nutritional needs in these patients are higher than what are currently recommended and applied. Jager‐Wittenaar et al.^67^ reported that patients with HNC undergoing treatment with an intake of >35 kcal/kg/day and >1.5 g protein/kg/day lost significantly less body weight and lean mass than did those patients consuming <35 kcal/kg/day and <1.5 g protein/kg/day. Furthermore, anabolic and anti‐catabolic strategies like exercise and specific nutrients (e.g. ω−3 fatty acids) or drugs are not applied in current practice.

The decrease in FFM was endorsed by a parallel significant decrease in HGS. This is in line with the study by Arribas et al.,^68^ but in contrast with Cosway et al.,^69^ who did not find a decreased HGS between start of therapy and 3 months post‐treatment. However, in the latter study, only weight loss was reported without any information on body composition.

Atasoy et al.^57^ did not find changes in lean body mass and body FM during CRT, and Isenring et al.^70^ found a trend towards increased FFM in the nutritional intervention group compared with usual care. Differences in nutritional intervention strategies might explain this dissimilarity.

In order to improve the decision and timing of TF administration and feeding tube insertion, the development of a decision model and subsequent nomogram on prophylactic gastrostomy insertion is in progress.

In the present population, the amount of weight loss throughout the course of CRT/BRT was not significantly related to a worse OS. Strikingly, literature provides divergent results as two studies^71^, ^72^ found that patients with increased weight loss showed better OS outcomes than did patients who gained weight during treatment, referring to the obesity paradox. Contrary to these findings, Ghadjar et al. reported a decreased OS in those who lost weight during CRT.^41^ Unfortunately, these study populations are quite heterogeneous, complicating definite analysis and also the identification of prognostic subgroups. Additionally, the relatively small study populations might also have influenced the reliability of the results.

The current study has some limitations. The analysis revealed several statistically significant results; however, the sample size was probably too small to allow detailed group stratification to detect all relevant relations. Nevertheless, the population of included patients was a realistic representation of HNC patients receiving CRT/BRT for LAHNSCC, which gives insight in the overall severity of muscle wasting, body composition, and OS in this group. Furthermore, measurements on HGS and BIA were collected prospectively according to the standardized protocol. However, information on TF was collected retrospectively, and therefore, the results of this study might be prone to (selection) bias. Specification of TF and actual amount administered could not be traced. Another limitation is the minimal dietary information on the normal diet, the dietary enrichment, and ONS in the TOD group. No exact records were kept of these specific items. Co‐morbidities were not reported, so the impact of this confounder on group differences between CRT and BRT receivers could not be confirmed nor ruled out.

Whole‐body magnetic resonance imaging and computed tomography (CT) are considered the gold standards in measuring body composition.^73^ Because whole‐body CT scans are not part of standard practice, these whole‐body CT scans were not available in the present cohort for body composition evaluation. Determining FM and muscle mass on slices of CT scans, a derivative of whole body CRT,^74^ was not preferred, because it could not provide information on the different body composition profiles. BIA measurement, a convenient and non‐invasive technique,^75^, ^76^ in combination with BMI enables researchers to verify these profiles and was therefore considered appropriate in this research.

## Conclusion

Muscle wasting is common in LAHNSCC, as nearly 30% of the present population undergoing CRT/BRT had muscle wasting at the start of CRT/BRT. Additionally, FFMI < P_10_ is an unfavourable prognostic factor for OS, treatment toxicity, and tolerance. Patients experience significant weight and FFM loss during treatment. The current TF regime attenuates weight loss but does not overcome loss of muscle mass and function during therapy. Future interventions should consider proactive monitoring of risk factors for muscle wasting, nutritional support tailored to reach the energy and protein requirements of the patients, and specific anabolic and anti‐catabolic nutrients, together with additional strategies targeting metabolism, loss of muscle mass, and function.

Further work should focus on the potential contributing factors, both intake dependent and metabolic drivers of muscle wasting, to allow for early identification of (pre)cachexia and personalized treatment strategies.

## Conflict of Interest

As indicated in the affiliations, A.V.H. is employed by Danone Nutricia Research. A.C.H.W., A.H., R.I.L., L.W.J.B., F.W.R.W., F.H., and A.M.W.J.S. declare that they have no conflict of interest.

## Appendix A.

**Table A1:** Baseline characteristics—cisplatin vs. cetuximab

Variables	Baseline group (*n =* 137)		
	Cisplatin (*n =* 100)	Cetuximab (*n =* 37)	*P*‐value
Age (years)	58.3 ± 7.9	62.0 ± 5.9	**0.010** a
Sex			
Male	73 (73)	20 (55)	
Female	27 (27)	17 (45)	**0.035** b
BMI (kg/m^2^)	24.9 ± 4.4	24.5 ± 5.9	0.629^a^
Mean prediagnostic weight			
loss (%)	2.4 ± 3.6	4.3 ± 6.3	**0.027** a
FFMI			
Normal FFMI	77 (77)	20 (54)	**0.009** b
FFMI < P_10_	23 (23)	17 (46)	
Patient‐reported OD at start RT			
Yes	20 (20)	17 (46)	
No	80 (80)	20 (54)	**0.002** b
Tobacco use			
Yes	88 (88)	37 (100)	
No	12 (12)	0 (0)	**0.027** b
Alcohol consumption of at least 1 per day			
Yes	54 (54)	27 (73)	
No	46 (46)	10 (27)	**0.045** b
WHO performance status			
0	17 (17)	3 (8)	
1	81 (81)	31 (84)	
2	2 (2)	3 (8)	0.119^b^
Handgrip strength ( kg)			
Male	46 ± 11	41 ± 11	0.122^a^
Female	29 ± 8	23 ± 5	**0.010** a
Primary tumour site			
Nasopharynx	7 (7)	0 (0)	
Oropharynx	37 (37)	16 (43)	
Hypopharynx	14 (14)	5 (14)	
Oral cavity	19 (19)	3 (8)	
Larynx	20 (20)	11 (30)	
Unknown primary	2 (2)	1 (3)	
Other	1 (1)	1 (3)	0.361^b^
T classification			
Tx	3 (3)	1 (3)	
T0	5 (5)	0 (0)	
T1	12 (12)	3 (8)	
T2	21 (21)	5 (14)	
T3	24 (24)	13 (35)	
T4	35 (35)	15 (41)	0.480^b^
N classification			
N0	18 (18)	8 (22)	
N1	15 (15)	1 (3)	
N2	64 (64)	27 (73)	
N3	3 (3)	1 (3)	0.259^b^
Tumour stage			
Stage II–III	20 (20)	4 (19)	
Stage IV	80 (80)	33 (81)	0.209^b^
P16			
P16+ oropharynx	22 (22)	5 (14)	
Others	77^c^ (78)	32 (86)	0.257^b^
CRT timing			
Primary	74 (74)	31 (84)	
Adjuvant	26 (26)	6 (16)	0.229^b^
Radiotherapy on neck			
Unilateral	7 (7)	1 (3)	
Bilateral	93 (93)	35 (95)	
No neck RT	0 (0)	1 (3)	0.168^b^
Tube feeding administration			
Yes	69 (69)	19 (51)	
No	31 (31)	18 (49)	0.056^b^

Bold values denote statistical significance at the *P* < 0.050 level.

BMI, body mass index; CRT, chemoradiation; OD, oropharyngeal dysphagia (Common Terminology Criteria for Adverse Events grade 2 OD or higher); RT, radiotherapy. Tumour, nodes, and metastasis (TNM) classification 7th edition^78^; WHO, World Health Organization.

a Independent samples *t*‐test.

b *χ* ^2^.

c One missing.

**Table A2:** Mean loss of masses and function during chemoradiation, tube feeding vs. total oral diet

	Tube feeding (*n* = 48)		Total oral diet (*n* = 21)		Between groups	
	Loss in kg	*P*‐value^a^	Loss in kg	*P*‐value^a^	Mean difference	*P*‐value^b^
Mass loss Week 1–4 of CRT in kg						
W	1.3 ± 2.6	**0.002**	1.4 ± 3.3	0.062	0.2 ± 0.7	0.828
FM	0.9 ± 3.1	**0.044**	0.3 ± 2.1	0.457	−0.6 ± 0.7	0.438
FFM	0.3 ± 3.2	0.474	1.1 ± 3.0	0.112	0.7 ± 0.8	0.376
Mass loss Week 4–end of CRT in kg						
W	1.7 ± 2.8	**<0.001**	4.0 ± 3.3	**<0.001**	2.3 ± 0.8	**0.004**
FM	0.8 ± 3.5	0.112	2.1 ± 3.3	**0.008**	1.3 ± 0.9	0.151
FFM	0.9 ± 3.2	0.054	1.9 ± 4.4	0.064	1.0 ± 0.9	0.298
Mass loss from start to end of CRT in kg						
W	3.0 ± 3.2	**<0.001**	5.5 ± 3.7	**<0.001**	2.5 ± 0.9	**0.007**
FM	1.7 ± 2.7	**<0.001**	2.5 ± 3.9	**0.008**	0.7 ± 0.8	0.373
FFM	1.2 ± 3.3	**0.012**	3.0 ± 4.3	**0.005**	1.7 ± 1.0	0.075
Loss of handgrip strength						
Start–Week 4	1.7 ± 4.5	**0.013**	1.8 ± 5.1	0.132	0.1 ± 1.2	0.948
Week 4–end	1.5 ± 5.2	0.057	1.3 ± 5.4	0.291	−0.2 ± 1.4	0.901
Start–end	3.1 ± 5.4	**<0.001**	3.0 ± 7.2	0.067	−0.0 ± 1.6	0.981

Bold values denote statistical significance at the *P* < 0.050 level.

Add‐up values from rows can slightly differ owing to rounding off to one decimal point.

CRT, chemoradiation; FFM, fat‐free mass loss; FM, fat mass loss; W, total weight loss.

a Paired samples *t*‐test.

b Independent samples *t*‐test.

**Table A3:** Dose‐limiting toxicity in cisplatin subgroup specified—muscle wasted vs. non‐wasted

Variables	Normal FFMI *n =* 77 (77%)	FFMI < P_10_ *n =* 23 (23%)	*P*‐value
Neutropaenia	10/77 (13%)	1/23 (4%)	0.245
Renal failure	4/77 (5%)	5/23 (22%)	**0.015**
Ototoxicity	6/77 (8%)	6/23 (26%)	**0.018**
Packed cells transfusion for anaemia	16/77 (21%)	8/23 (35%)	0.168

Bold values denote statistical significance at the *P* < 0.050 level.

FFMI, fat‐free mass index.
